# An innovative autonomous robotic system for on-site detection of heavy metal pollution plumes in surface water

**DOI:** 10.1007/s10661-021-09738-z

**Published:** 2022-01-24

**Authors:** Elisabetta De Vito-Francesco, Alessandro Farinelli, Qiuyue Yang, Bhawna Nagar, Ruslan Álvarez, Arben Merkoçi, Thorsten Knutz, Alexander Haider, Wolfgang Stach, Falko Ziegenbalg, Roza Allabashi

**Affiliations:** 1 Department of Water, Atmosphere, and Environment, Institute of Sanitary Engineering and Water Pollution Control, University of Natural Resources and Life Sciences Vienna (BOKU), Muthgasse 18, 1190 Vienna, Austria; 2 Department of Computer Science, University of Verona, Ca Vignal 2, 37134 Verona, Italy; 3 Catalan Institute of Nanoscience and Nanotechnology (ICN2), UAB Campus, 08193 Bellaterra (Barcelona), Spain; 4 Materials Science, Department of Chemistry, Universitat Autònoma de Barcelona, Plaça Cívica, 08193 Bellaterra (Barcelona), Spain; 5 Laboratory of Physical and Analytical Electrochemistry (LEPA), Ecole Polytechnique Fédérale de Lausanne (EPFL) Valais Wallis, Rue de l’Industrie 17, 440, 1951 Sion, Switzerland; 6 Catalan Institution for Research and Advanced Studies (ICREA), Passeig Lluís Companys 23, 08010 Barcelona, Spain; 7 Go Systemelektronik GmbH, Falunerweg 1, 24109 Kiel, Germany

**Keywords:** Heavy metal pollution, Surface water monitoring, Square wave anodic stripping voltammetry, Screen-printed electrode, Autonomous surface vehicle

## Abstract

Smart monitoring has been studied and developed in recent years to create faster, cheaper, and more user-friendly on-site methods. The present study describes an innovative technology for investigative monitoring of heavy metal pollution (Cu and Pb) in surface water. It is composed of an autonomous surface vehicle capable of semiautonomous driving and equipped with a microfluidic device for detection of heavy metals. Detection is based on the method of square wave anodic stripping voltammetry using carbon-based screen-printed electrodes (SPEs). The focus of this work was to validate the ability of the integrated system to perform on-site detection of heavy metal pollution plumes in river catchments. This scenario was simulated in laboratory experiments. The main performance characteristics of the system, which was evaluated based on ISO 15839 were measurement bias (Pb 75%, Cu 65%), reproducibility (in terms of relative standard deviation: Pb 11–18%, Cu 6–10%) and the limit of detection (4 µg/L for Pb and 7 µg/L for Cu). The lowest detectable change (LDC), which is an important performance characteristic for this application, was estimated to be 4–5 µg/L for Pb and 6–7 µg/L for Cu. The life span of an SPE averaged 39 measurements per day, which is considered sufficient for intended monitoring campaigns. This work demonstrated the suitability of the integrated system for on-site detection of Pb and Cu emissions from large and medium urban areas discharging into small water bodies.

**Supplementary Information:**

The online version contains supplementary material available at 10.1007/s10661-021-09738-z.

## Introduction

Water is of fundamental importance for the environment and life, and it determines the health of humans and animals (Silva Junior et al., 2016; Tuna et al., 2013). Both natural processes, such as weathering of soil, and anthropogenic activities, such as rapid industrialization, urbanization and agricultural activities, deeply influence the contamination of water resources with a variety and a large number of contaminants (Law et al., 2018; Li et al., 2015; Mohammed et al., 2016). In particular, there is great concern regarding the environmental fate of heavy metals (HMs) and their impacts on human health, which are related to bioaccumulation, non-biodegradability, persistence and toxicity (Gautam et al., 2014; Güell et al., 2008; Gumpu et al., 2015; Jang et al., 2011; Law et al., 2018; Li et al., 2015; Masindi & Muedi, 2018; Odobašić et al., 2019; Waheed et al., 2018). The ability of metals to pass through cell membranes causes several harm for different physiological processes of cells and therefore poses a threat to human and animal health (Gumpu et al., 2015; Jang et al., 2011). The majority of heavy metal releases into water bodies in Europe are related to urban pollution coming from urban wastewater treatment plants (UWWTPs, 45.5%), from misconnections and cross-connections, untreated combined sewer overflows or road runoff (EEA, 2018). Furthermore, the majority of the sources of heavy metals to these UWWTP come from industries (78.4%) (EEA, 2018). Heavy metals are considered indicators for the abovementioned emission sources, and their monitoring is of crucial importance in identifying the sources and pathways of potential pollutants in the aquatic environment.

Consistent and smart monitoring contribute to the preservation and protection of the aquatic environment and avoid negative effects on human health (EEA et al., 2018; EU, 2000, 2013; Melo et al., 2019; Odobašić et al., 2019). The traditional monitoring methodology consists of discrete or cumulative, manual or automatic sampling, followed by laboratory analysis (Berho et al., 2009; EEA, 1996; Steccanella et al., 2019). Within this monitoring process, sampling and laboratory analysis must be included. The most commonly used analytical methods for heavy metal analysis are inductively coupled plasma mass spectrometry (ICP-MS), inductively coupled plasma atomic emission spectrometry (ICP-AES), atomic absorption spectroscopy (AAS), and atomic fluorescence spectroscopy (AFS) (Gumpu et al., 2015; Koller & Saleh, 2018; Verma & Singh, 2005). These conventional techniques achieve high accuracy and sensitivity in measurements, but require a sampling procedure and a well-trained analyst. Furthermore, they are time-consuming and expensive (Berho et al., 2009; Butterfield, 2009; Gumpu et al., 2015; Verma & Singh, 2005).

Faster, less expensive and more user-friendly on-site methods are increasingly playing a crucial role in the field of quality monitoring of surface water bodies (Berho et al., 2009). In this regard, electrochemical techniques are very promising due to their sensitivity, low cost, accuracy, simplicity and on-site applicability (Berho et al., 2009; Lu et al., 2018; Waheed et al., 2018). Voltammetry, particularly square wave anodic stripping voltammetry (SWASV), is the technique usually applied to electrochemical measurements. Its virtues, relative to other techniques, include higher sensitivity, selectivity, shorter detection time and lower costs (de la Escosura-Muñiz et al., 2008; Güell et al., 2008; Lu et al., 2018; Merkoçi et al., 2000; Waheed et al., 2018). Printing technique, as massive production with low cost, has brought the fabrication of micro- and nano- sensors out of cleaning rooms and has been harnessed widely in sensing applications especially in stack structure (Wiklund et al., 2021). However, due to the difficult manipulation of different inks to be printed on the stack structure, fully printed sensors and sensing systems are still an open challenge (Khan et al., 2020). To avoid the limitations associated with bulky electrodes usually employed for voltammetry, the use of fully screen-printed electrodes has recently been applied for on-site analysis (Berho et al., 2009; Güell et al., 2008; Waheed et al., 2018). An additional positive feature of anodic stripping voltammetry for environmental monitoring is its ability to detect the soluble ionic form of metals (Barón-Jaimez et al., 2013; Zinoubi et al., 2017), which is the fraction responsible for metal bioavailability in the aquatic environment (Rensing & Maier, 2003); this quantity is targeted in the field of water policy for regulation of priority substances (EU, 2000, 2013) in the framework of the European Water Frame Directive (EU, 2000).

To overcome issues arising from conventional monitoring methods, including the high costs and lack of information between sampling campaigns, an alternative monitoring method involves, in addition to fixed monitoring stations, the use of autonomous surface vehicles (Steccanella et al., 2019). These systems consist of mobile, autonomous robotic systems, which can be programmed to perform activities independently; for instance, they can engage in sampling or real-time/on-site analysis and measurements of the matrix to be monitored (Melo et al., 2019).

The need for new monitoring concepts for detection of pollutants in surface bodies is included in the vision of the Horizon 2020 European-funded project INTCATCH. The project emphasizes the need to create smarter monitoring concepts using innovative technologies and holistic concepts. The present paper describes one of the monitoring systems developed within that framework, which consists of an autonomous surface vehicle equipped with a microfluidic electrochemical heavy metal detection device, named integrated system. The main aim of the present study is to demonstrate that this integrated system is fit-for-purpose in monitoring wastewater point emissions in surface waters, which means it is capable of detecting heavy metal pollution plumes in surface waters coming from wastewater spill-outs. Lead and copper were selected as target metals for system validation. Lead is included in the list of priority substances from the Water Framework Directive (WFD) (EU, 2000, 2013), and copper is a known representative of the pollutants released into water bodies from mining activities (EEA, 2018), industry (EEA, 2018) and stormwater runoff (Gromaire et al., 2001; Müller et al., 2019; Winters & Graunke, 2014; Winters et al., 2015). The present study first describes the components of the integrated system: the autonomous surface vehicle and the HM detection device. Second, a comprehensive validation involving laboratory and field experiments is performed to establish the performance characteristics of the system. The validation was carried out based on the guidelines available in the ISO 15839 standard (International Standard, 2003, 2019). The validation process included the evaluation of the integrated system during the simulation of real scenarios in the laboratory facility, followed by on-site campaigns to test the simultaneous operation of autonomous navigation and measurement. Finally, the validation results are further discussed in terms of the fit-for-purpose status of the integrated system, which depends on its ability to detect the impact of certain pollution sources on receiving waters.

## Material and methods

### Autonomous surface vehicle

**Fig. 1:**
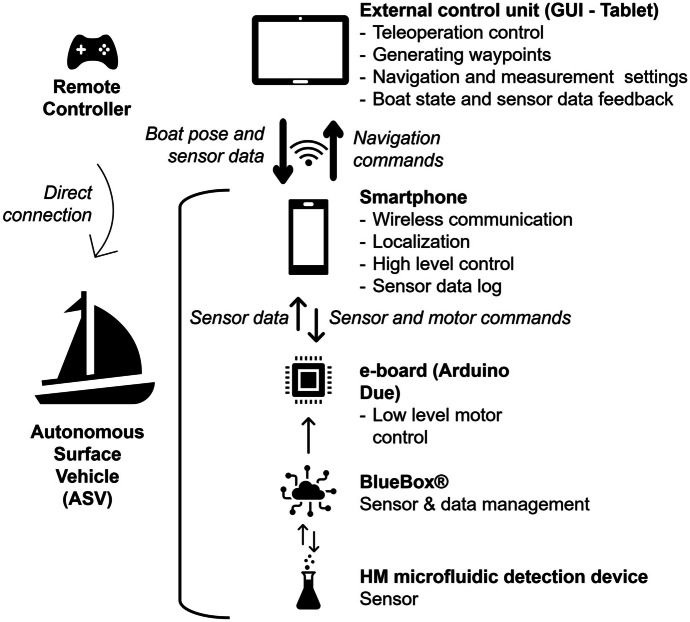
Schematic representation of the architecture of the autonomous surface vehicle’s control system, including the options for manual remote control (RC)

**Fig. 2:**
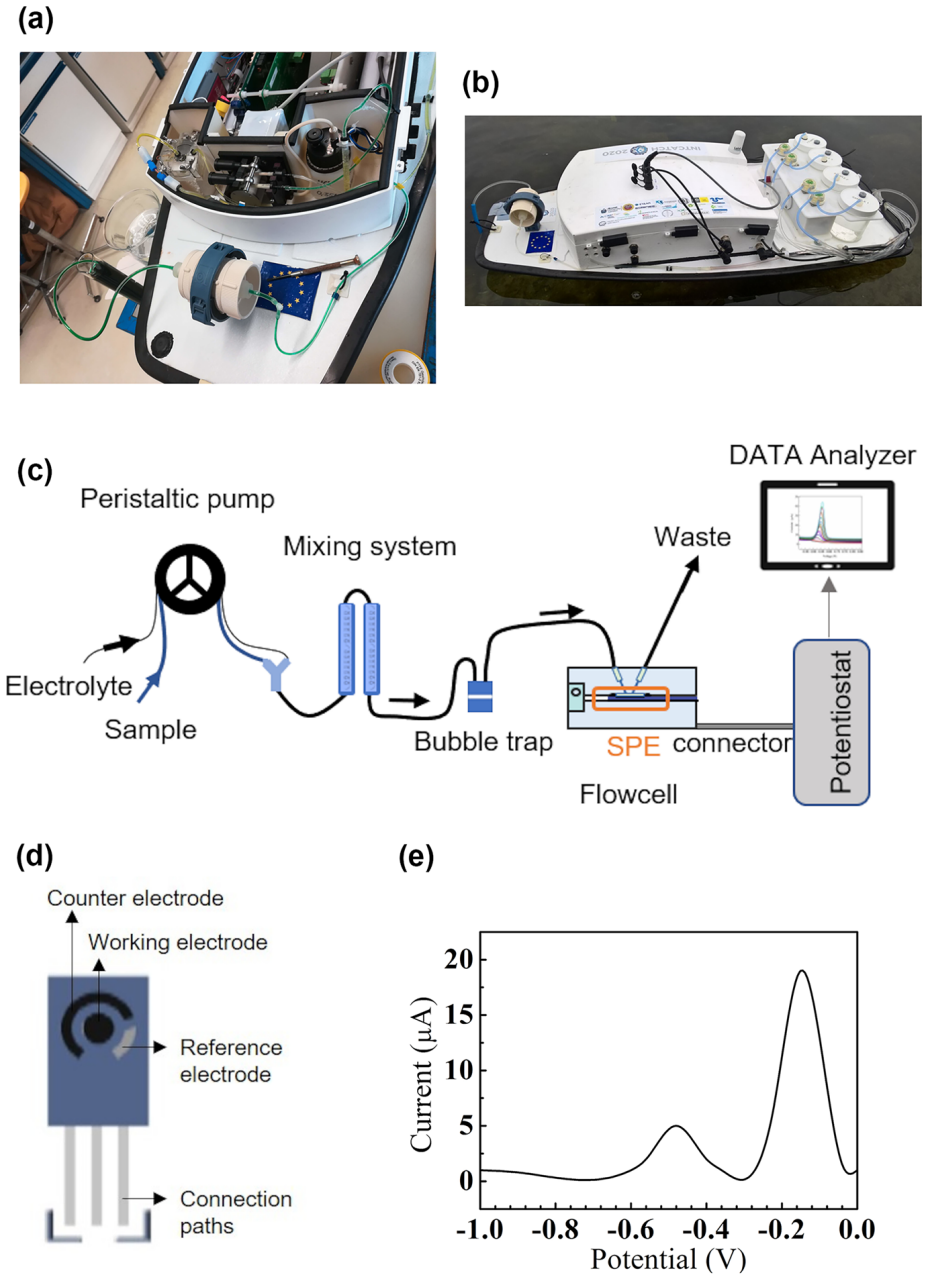
**a** Implementation of filtration system and HM microfluidic detection device on the autonomous surface vehicle; **b** autonomous surface vehicle, equipped with HM microfluidic detection device, filtration system, and sampler, deployed in a field experiment; **c** schematic representation of components of the HM microfluidic detection device; **d** schematic representation of one SPE; **e** example of the voltammogram

In the aforementioned project INTCATCH 2020, a commercial model of the autonomous surface vehicle (ASV; Fig. 2b) Lutra Prop®, was used. This is a monohull boat mounted with submerged propellers and measuring approximately 1 m long and 0.5 m wide, and it was developed by Platypus® (Steccanella et al., 2019). The platform has been engineered and optimized within the INTCATCH project to include several additional sensors and advanced autonomy capabilities. The main components of the architecture of the electronic system are an e-board, a measurement and sensor manager (Bluebox®), a smartphone and an external control unit device (tablet). A schematic representation of the architecture of the boat is shown in Fig. 1. The architecture of the ASV allows the end user to control the navigation feature settings remotely, start on-site measurements and start the sampling of the monitored matrix. The boat is powered by one lithium polymer battery (4 S, 16 Ah, 10 C, 16–14 V).

Incoming and outcoming control signals for the autonomous surface vehicle are managed by the e-board. It is the core of the autonomous surface vehicle and is composed of a Platypus circuit board and an Arduino Due® board. It connects to and provides power for all the other components. BlueBox® is a measurement data manager that manages the interpretation of the measured electrical signals and is directly connected to the implemented measuring device. Furthermore, it uploads and stores the measurement data in the Cloud Database and locally on the Bluebox® system. The smartphone is used as the main computation component of the ASV, controlling its autonomous functions and receiving navigation and measurement commands via a WiFi connection with an external control unit (tablet). The latter is equipped with a graphical unit interface (GUI). Through the GUI, the end-user can load live maps of the selected catchment and therefore to visualize the position of the ASV and user. It is possible to send commands to control navigation features (customizable design of waypoints, pathways and velocity levels) and the measuring system device. With this option, the end user can initiate the HM measurement by choosing between “single” and “continuous measurement” modes. The first consists of the performance of a single measurement, and the second comprises consecutive and continuous repetition of single measurements. Another possibility offered by the GUI is the autonomous sampling of the monitored matrix (Fig. 2b). This consists of the independent filling of four sampling containers integrated into the autonomous surface vehicle (0.5 L volume each). These water samples are needed first for heavy metal reference analysis and second for analysis of additional parameters to complete the water quality information.

### Heavy metal detection device

The autonomous surface vehicle is equipped with a microfluidic detection device to provide for HM on-site detection (Fig. 2a). This system consists of a peristaltic pump, an automatic mixing system, a bubble trap, a flow cell and a potentiostat (Fig. 2c). A carbon paste screen-printed electrode (SPE), including a miniaturized three-electrode electrochemical system as the main sensing element, is fixed in the flow cell and connected with the potentiostat (Fig. 2d). In comparison with a three-electrode system, in a two-electrode system the reference and the counter electrodes are merged into one electrode. In this case, it is more difficult for the reference electrode to provide a stable reference potential and simultaneously collect electrons as counter electrodes. Therefore, the three-electrode electrochemical system was selected as suitable for the integrated system. The microfluidic detection device is compatible with the electrochemical method of square wave anodic stripping voltammetry (SWASV), generally coupled with a three-electrode electrochemical system. First, through the application of a specific negative potential on the working electrode, the HM ions are concentrated, accumulated and reduced with the flow passing through the flow cell and over the SPE. Subsequently, the potential is released to 0 by square waves used to strip back the HM ions. The raw data, or voltammogram, consist of a plot of the measured electrical current obtained from HM ions stripping (mA) as a function of the potential (V). The various peak potentials are assigned to different heavy metal species, as in the example shown in Fig. 2e), where the first peak corresponds to Pb and the second to Cu. The intensity of the current signal is proportional to the concentration of the analytes in the sample. In our study, the integrated area below the electrical current peak (µAV) was used as the output signal. The HM microfluidic detection device does not perform sample stirring or any pretreatment of the samples other than filtration (Fig. 2a) and mixing with the electrolyte needed for analysis. The sample is filtered through two in-sequence nylon net (Millipore) filters with 180 µm and 80-µm net pores and of 47 mm diameter. The presence of an electrolyte (HCl) increases the release of metal ions present in the water sample from bound forms and helps with the transportation of ions during the measurement. The ratio of the sample to electrolyte was set to 7:1 to obtain a final concentration of 0.05-M HCl.

Since the integrated system is still a proof of concept as a fully automatic tool to simultaneously detect multi-HMs, lead and copper were chosen as model HMs and target heavy metals for the system validation. During the experiments performed to validate the detection of all representative heavy metal pollutants, such as zinc (Zn), cadmium (Cd), lead (Pb) and copper (Cu), it turned out that Cd presents a high limit of detection (LOD) due to the mutual interference with other heavy metals and the potential for Zn (~ −1.2 V) does not fit in our SWASV sensing potential window/range (from −1.0 to 0 V). Consequently, we focused on Pb and Cu for the purpose of this study. Any further details concerning the set-up and the sensing performance of the HM microfluidic detection device can be found in the publication from Yang et al. (2021).

### Measurement and quality assurance

The raw data of a single measurement performed with the HM device consisted of the measured electrical current related to the corresponding potential (Fig. 2e). The potentials at which electrical current peaks occurred and the integrated areas below those peaks were automatically computed by the software used. Before each experimental campaign, a fresh screen-printed sensor was employed and calibrated. Representative voltammograms are shown in Fig. 10 in the Supporting information.

The calibration curves were generated from measurements of mixed solutions of both metals, Pb and Cu, performed with the described integrated system (microfluidic detection device implemented on the ASV). The calibration solutions were first prepared within a concentration range of 10–100 µg/L, keeping the 1:1 ratio for both metals. For a better representation of the concentration ratio of these metals in real surface waters, some experiments were performed with mixed solutions containing a 1:4 ratio (Pb:Cu), leading to a working range of 40–400 µg/L for Cu. Two kinds of linear regression functions, linear forced and non-forced through zero and a non-linear regression function, were considered regarding their influence on the measuring accuracy.

To perform quality assurance of the measurements, mixed control samples (CS) of Pb and Cu were prepared with a concentration at the middle of the defined working range. The control samples were prepared in the lab from stock solutions (1000 mg/L Pb, as Pb(NO_3_)_2_ in H_2_O, Titrisol®, 1.09969.0001 and 1000 mg/L Cu, as CuCl_2_ in H_2_O, Titrisol®, 1.09987.0001, VWR). Both calibration solutions and control samples were always measured with the microfluidic detection device implemented in the integrated system.

### Validation procedure

The methodology used to validate the integrated system was based on the ISO 15839 protocol (reviewed in 2019) “Water quality – online sensor/analysing equipment for water – specifications and performance tests” (International Standard, 2003, 2019). The named standard was reviewed and confirmed in 2019; therefore, the current version can be considered to be used.

This standard can be applied to most sensors/analysis equipment, but it is recognized that for some equipment, certain performance tests cannot be carried out (International Standard, 2003, 2019). Therefore, the validation procedure for the equipment under study (integrated system) was focused on the assessment of performance characteristics, which were especially useful to describe the suitability of the system for the intended purpose. As the intended purpose is the detection of heavy metal pollution plumes in surface waters, the most important performance characteristic is considered to be the lowest detectable change (LDC), which is defined as the “smallest significantly measurable difference between two measurements”. This characteristic gives information about the ability of the measuring system to detect spatial concentration gradients in a defined area around the pollution source. Additionally, the limit of detection (LOD) and limit of quantification (LOQ) were evaluated according to the test procedure described in ISO 15839. Day-to-day repeatability was chosen to address measurement precision. The evaluation of systematic error (bias) was performed mainly through recovery experiments, which involved the extent of recovery of a known amount of Pb and Cu added to a previously analysed sample (International Standard, 2012). A comparison of measured values with two selected reference methods was also performed for this purpose and discussed in the section “Reference analysis”. The data acquired from the laboratory experiments were used to calculate the aforementioned performance characteristics.

### Laboratory experiments

**Fig. 3:**
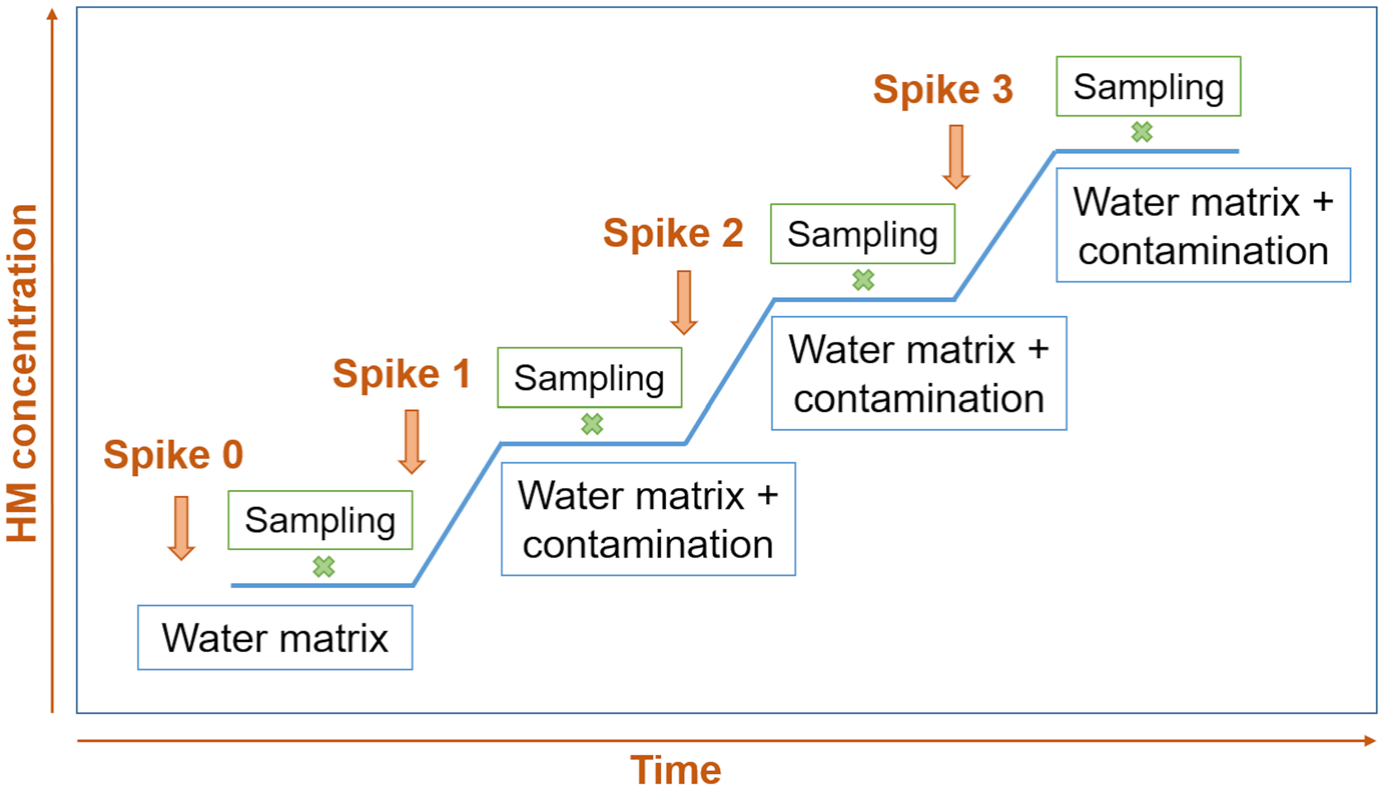
Schematic representation of the laboratory experimental procedure

Laboratory experiments were designed and performed to validate the integrated system for use with the case of a potential spill-out of polluted wastewater into a surface water body. This situation was simulated in a laboratory set-up and is shown in Fig. 3. A 500-L water tank was filled with water collected from the Danube River or from a groundwater well, which represented bank-filtrated Danube River water. The introduction of prepared solutions of lead (1000-mg/L Pb, as stock solution) and copper (1000-mg/L Cu, as stock solution) into the real water matrix was used to simulate point-source contamination. The matrix was spiked at defined time intervals of approximately 60 min (Fig. 3). The desired theoretical concentrations for each spike level, as in the example shown in Fig. 6, were 25, 50, 75 and 100 µg/L for Pb and 100, 200, 300 and 400 µg/L for Cu. The number of spiking levels and the desired concentrations varied slightly between experiments. To avoid the sedimentation of suspended particles, a stirrer was situated within the tank and operated at 1000 rpm. The integrated system was located in the tank and programmed to perform measurements in continuous mode during the whole simulation. For the reference analysis of Pb and Cu in the lab, duplicates of samples were collected 30 min after each contamination spike.

### Reference analysis

Photometry was identified as the proper reference method for comparison and calculation of recovery because of its ability to analyse free ions in water, as well as the SWASV method, used in the integrated system. Therefore, similar results are expected between the photometric method and the SWASV one. Furthermore, the photometry method was considered for its portability, and therefore a potential on-site reference method. The kits “copper trace cuvette test” and “lead trace cuvette test” (Hach Lange GmbH¸ LCK529 and LCK306, respectively) were used to perform the reference analyses without any pretreatment of the water samples. The bathocuproine disulphonic acid method and the PAR method (4-(2-pyridylazo)-resorcinol) were applied for Cu and Pb, respectively. The working ranges for these kits are 0.01–1.0 mg/L and 0.1–2.0 mg/L for Cu and Pb, respectively (HACH, ﻿^2019^, ^2020^).

Even though inductively coupled plasma mass spectrometry (ICP-MS) is known to analyse the total amount of heavy metals in water, therefore less feasible than the photometric method, it was additionally selected as a reference method for two reasons. First, in the integrated system, the sample was premixed with acid prior to SWASV measurement so that a certain amount of ions were released from their bound state into the free state (acidifying process (Güell et al., 2008; Palchetti et al., 2005)). In this case, the content of metal ions measured was expected to be higher than that measured by the photometric method. Therefore, depending on the release rate, the quantity measured by the integrated system (SWASV) could be better compared to that of ICP-MS. Second, the chosen photometric method fails to measure concentrations smaller than 0.01 and 0.1 mg/L for Cu and Pb, respectively. In these cases, ICP-MS was the only possible analytical method available as a reference analysis. The instrument Elan DRC-e (PerkinElmer Inc.) was used to perform this analysis by applying the standard methods DIN EN ISO 17294–1 (E36)-2003 for the application of the ICP-MS and DIN EN ISO 17294–2 (E 29)-2004 for the selected metals. The limits of quantification (LOQs) of the method used were 0.5 µg/L and 1.0 µg/L for Pb and Cu, respectively.

### Field experiments

Field experiments consisted of deploying the integrated system at a selected location and testing its abilities in a real environment. The main goal of the performed field experiments was to test the simultaneous functioning of the main features of the integrated system while the ASV was set to autonomous navigation; these involved continuous measurements with the HM microfluidic detection device and automatic filling of the vessels with the automatic sampler device.

The selected site was located within the City of Vienna, at the New Danube River, in the vicinity of an oil refinery (48° 11′ 09.8″ N 16° 28′ 37.1″ E). Prior to deployment, the calibration of the screen-printed sensor was performed in the laboratory.

The set-up of the integrated system was made possible with the GUI. The speed was set at “low speed”, which corresponded to ~ 1 m/s. The path (waypoints) was designed to cover an area of ~ 100 × 15 m of the water body. The HM detection device was set to “continuous measuring mode”. The measurement time was established to be approximately 2 h for each deployment. Each single HM measurement lasted ~ 7 min, therefore allowing ~ 17 on-site measurements. The values measured were stored in Bluegate (the cloud of the Bluebox® data manager).

In addition, the collection of four water samples for each campaign was possible with the integrated sampling device, as described in the “Autonomous surface vehicle”.

## Results and discussion

### Calibration function

The analysis of Pb and Cu using screen-printed electrodes (SPE) coupled with SWASV was based on a calibration function generated prior to sample analysis. The values of the measured peak areas (µAV, two representative voltammograms are included in Fig. S1) for the selected calibration solutions were plotted in relation to the known concentration values of these solutions. The calibration function was then obtained by fitting the plotted points with a regression trend. As the calibration function strongly influenced the accuracy of the final measurement, its role was first evaluated. In the case of SWASV, the limited lifetimes of SPEs made this crucial, as the calibration function related to each specific SPE had to be changed daily for new experiments and/or measuring campaigns.

Three regression models were considered for the calibration function: linear, linear forced through zero and non-linear (polynomial of second order). Using a large number of calibration functions performed (40 in total), it was observed that all three calibration models exhibited good fits, with *R*^2^ > 0.90 for most cases. Even though the polynomial model showed better values (*R*^2^ > 0.98), fits with both linear calibration models were considered satisfactory for this application. Therefore, the linear regression model was selected for further computations. The following labels were used throughout the study: “cal1” indicated the calibration function obtained with linear regression, and “cal2” indicated the calibration function obtained with linear regression forced through zero. It was further observed, as expected, that the higher intercepts of calibration functions (cal1) led to inaccurate values at low concentrations of heavy metals in samples. The authors decided to use the forced-through-zero calibration function (cal2) for further calculations; although this is not the way to improve measurement accuracy, it is a reasonable compromise for the analytical method used and the purpose of this study.

**Fig. 4:**
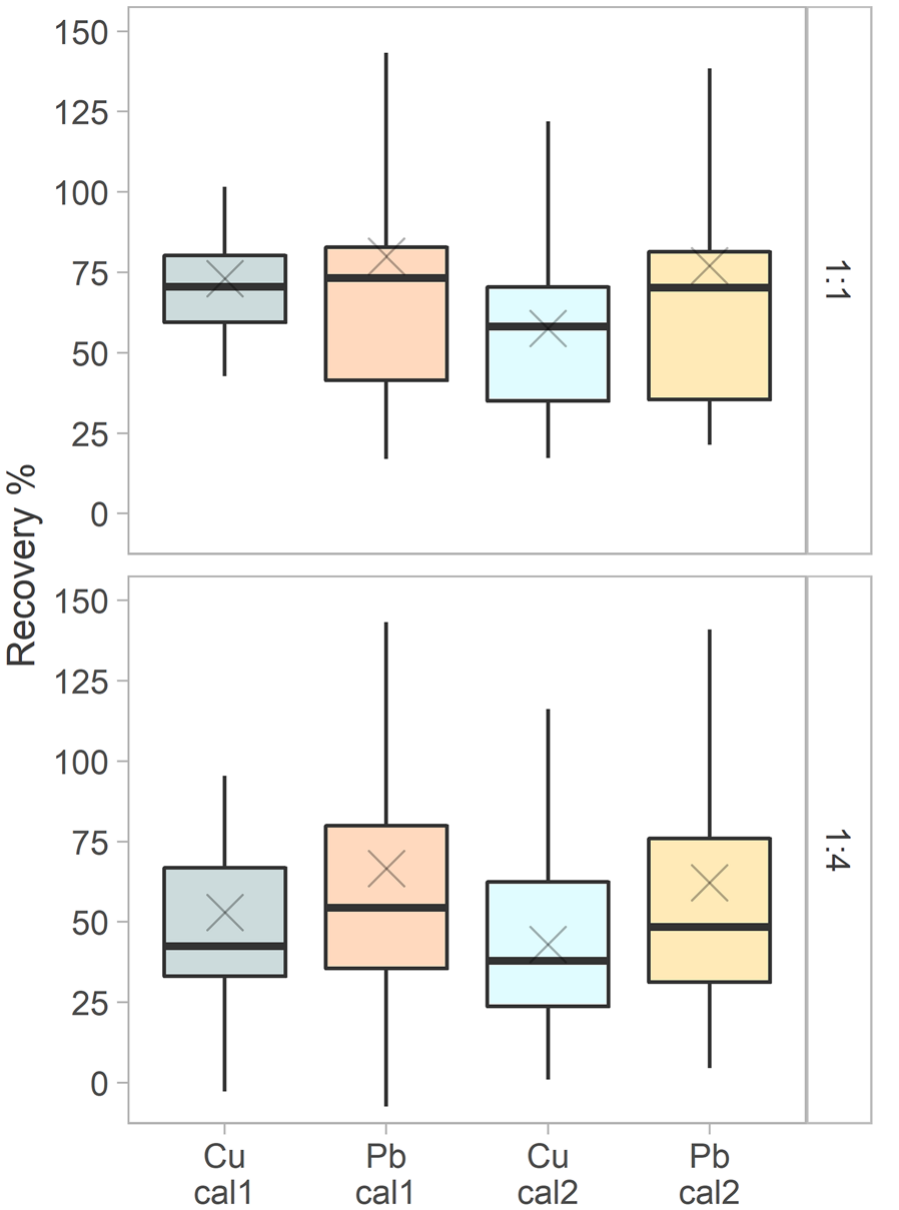
Recovery of the control sample (CS) for Pb and Cu and for ratios of 1:1 and 1:4, calculated with both linear calibrations cal1 (linear regression) and cal2 (linear regression forced through 0). The mean values of the recoveries are represented by the cross symbols

In Fig. 4, the recovery (%) of the control samples for Pb and Cu, computed with both cal1 and cal2, is shown. The recovery for Pb was between 70 and 73% (median values, ratio 1:1) and 48 and 54% (median values, ratio 1:4), and for Cu it was between 58 and 70% (median values, ratio 1:1) and 38 and 42% (median values, ratio 1:4). The selection of the calibration function did not have a significant influence on the accuracy of Pb and Cu measurements. This can be explained by the fact that the concentrations of control samples were selected to be in the middle part of the calibration range, where the influence of the changed intercept is expected to be minimal.

The authors are aware that both characteristics, the linearity of the calibration function and the accuracy in terms of control sample recovery, are rather poor compared to those of standardized laboratory methods. However, they can be considered acceptable for the aforementioned compromise between on-site sampling application in combination with the purpose of this study and the detection of wastewater source-point emissions in surface water. In such cases, a pollution plume is expected, and the most important performance characteristic for detection is considered to be the lowest detectable change, rather than measurement accuracy.

### Sensor lifetime

**Fig. 5:**
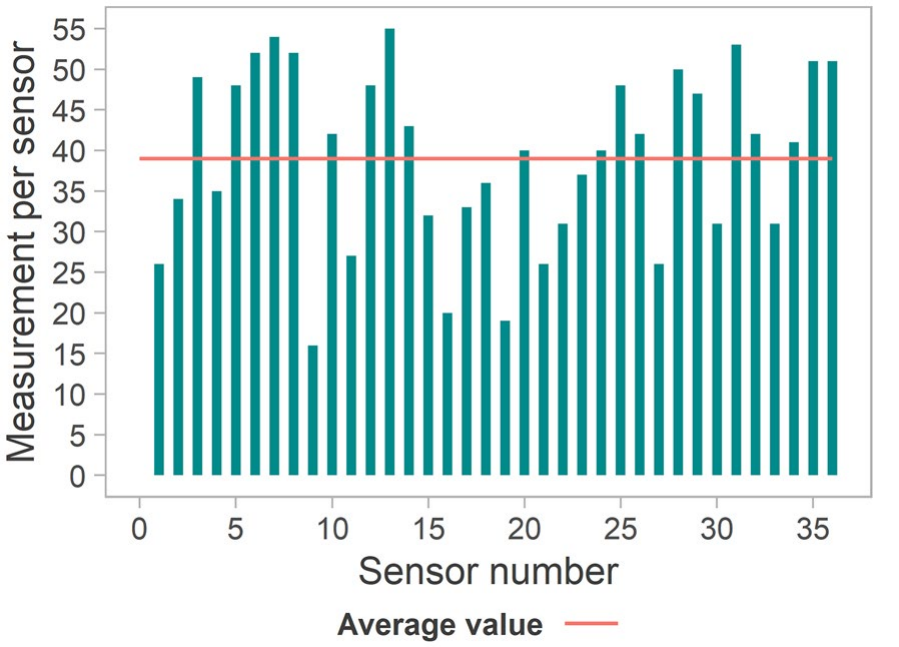
Number of measurements performed with each SPE and average value and average value represented by the horizontal line

The main evaluation of the analytical performance of SPEs under laboratory conditions was already performed by the system developers and is reported in Yang et al. ﻿(2021). In the context of this work, only the lifetime of the SPE involved in the integrated system and in use during the measuring campaigns was observed and documented. This is considered an important factor for field monitoring campaigns because of the limited number of measurements allowed per SPE and the necessity to use a fresh SPE for each measuring campaign (day). The results are shown in Fig. 5, where each bar shows the number of measurements performed with one SPE during an entire laboratory or field experiment, including the preliminary calibration. The maximum number of measurements performed with 1 SPE was 55, and the minimum number was 16. It must be outlined that these low values (16, 19, 20 measurements) were not related to poor quality or damaged sensors but simply to other technical issues arising during the experiments (e.g. malfunctioning of the integrated system during the experiment), which required a change of sensor. The calculated average value of 39 measurements per sensor, which included an initial quality assurance check (triple measurement), generation of the calibration function needed (triplicate measurement of five calibration solutions) and finally, effective monitoring of the water body, was considered sufficient for the intended monitoring campaigns.

### Validation through laboratory experiments

**Fig. 6:**
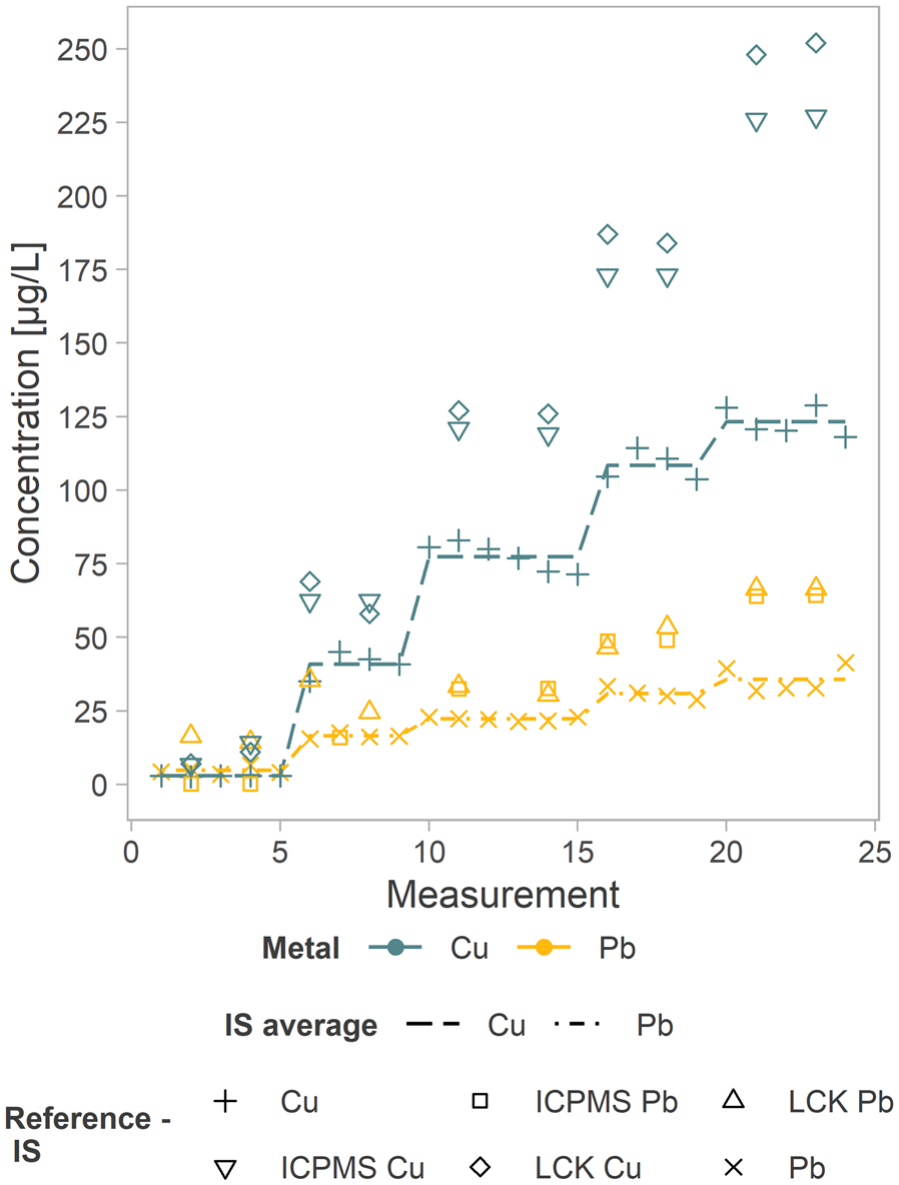
Laboratory experiment results, including lead (orange) and copper (blue) in a ratio of 1:4 and reference analyses

The experiment reported in Fig. 6 consisted of the simulation of four contamination levels in the range of 0–100 µg/L for Pb and 0–400 µg/L for Cu and use of bank-filtrated Danube River water as the matrix, and it is considered a representative example of seven similar laboratory experiments performed.

Figure 6 shows the Cu and Pb concentrations measured by the integrated system (IS) and computed with cal2, the mean concentration values for each spike level and results of reference analyses. Table 1 summarizes the results obtained during the indoor experiments. The difference between the mean concentration values of consecutive levels should be constant, as the water matrix was spiked with the same amount of contaminants at each level. For Cu, the concentration differences of consecutive levels showed a rather constant behaviour for the first three levels (~ 35 µg/L differences, instead of the theoretical difference of 25 µg/L) and some discrepancy for the last level (only ~ 14 µg/L increase). Severe variability was observed for Pb, in which this difference decreased for each spike level. Nevertheless, the integrated system was able to detect the change in concentration for both metals at each contamination level.

**Table 1:** Indoor experiment: average (AV) concentrations (conc.) detected by the integrated system (IS) and by the two reference methods (ICP-MS and LCK) and recoveries relative to the theoretical (theor.) value and the measured reference values (ICP-MS and LCK). Values in brackets are values < LOQ

	**Theor. concen-tration**	**AV conc.**	**AV conc.**	**AV conc.**	**Recovery (theor. conc.)**	**Recovery (ICP-MS)**	**Recovery (LCK)**
		**IS**	**ICP-MS**	**LCK**			
Unit	µg/L	µg/L	µg/L	µg/L	%	%	%
**Pb**							
Level 1	0.0	**(4.7)**	< 0.5	(15.5)	-	-	-
Level 2	25.0	**16.4**	15.9	30.0	66	103	55
Level 3	50.0	**22.2**	32.6	32.0	44	68	70
Level 4	75.0	**30.8**	48.9	50.0	41	63	62
Level 5	100.0	**35.6**	64.2	66.5	36	56	54
**Cu**							
Level 1	0.0	**(2.9)**	10.5	(9.0)	-	-	-
Level 2	100.0	**40.9**	62.5	63.5	41	65	64
Level 3	200.0	**77.4**	120.0	126.5	39	65	61
Level 4	300.0	**108.4**	173.0	185.5	36	63	58
Level 5	400.0	**123.2**	226.5	250.0	31	54	49

As seen in Table 1 and in Fig. 6, significant discrepancies were observed between measured values determined with the integrated system and reference methods. The calculated recoveries for Pb were 56–103% and 54–70% and those for Cu were 54–65% and 49–64%, relative to ICP-MS and LCK results, respectively. The reference methods showed similar values to each other while showing a discrepancy with the SWASV method applied to the integrated system. One possible explanation for this could be the existence of various metal species in the water matrix and the abilities of the analytical methods to address them differently.

The employed SWASV method detected the free metal ions soluble in water (Barón-Jaimez et al., 2013; Zinoubi et al., 2017), but not the fraction bonded in complexes and other stable chemical compounds. Most metals have the ability to form complexes with organic substances or other compounds present in the water matrix. Dissolved organic matter (DOM), in particular humic and fulvic substances, serves as the main source for organic ligands able to build stable complexes with most metal ions (Boggs et al., 1985; Mostofa et al., 2013a; Mostofa, 2013). These compounds exhibit a strong interaction with ion metals, forming complexes with covalent bonds (Boggs et al., 1985; Mostofa et al., 2013b; Pandey et al., 2000). These substances are always present in surface waters and, according to Mostofa et al. (2013b), comprise between 20 and 85% of the DOM in rivers. Humic substances are also the main component in DOM of groundwater environments (Pisarek & Głowacki, 2015; Steinberg et al., 2003). These substances also play an essential role in the complexation of metal ions in water, thereby decreasing their availability as free ions (Pisarek & Głowacki, 2015; Steinberg et al., 2003). Considering that the ICP-MS method provides the total ion concentration (free and complexed form), the discrepancy between the ICP-MS and integrated system results may be explained by the presence of metal complexes of humic or fulvic substances in the river water used for experiments. The gap between the concentrations measured with the integrated system and the ICP-MS reference method indicates that a certain part of Pb and Cu in the river water samples could not be released from the organic complexes before measurement, despite the pre-acidification with HCl. One possible reason for this may be the short contact time of HMs and HCl in the described micro fluidic detection device.

The photometric method was selected as a reference method based on its ability to detect free metal ions in water without any sample pretreatment; hence, it is expected to give results similar to those from the SWASV method. Nevertheless, a significant difference in detected concentrations was also observed between these two methods, with significantly lower results for SWASV. This could be attributed to two factors. First, the real sample matrix may influence the sensing capability through a possible deposition of DOM onto the surface of the working electrode during the deposition step. This would enhance the background current and block active sites, leading to a reduced signal for the heavy metal (Borrill et al., 2019; Lam et al., 1997). From our observation, the measurement baseline for Danube river samples kept shifting higher with an increase in the number of measurements, which may be caused by the described effect of DOM. Second, the lower results for the SWASV method could be related to the capability of the screen-printed electrode to collect and detect the free ions from water, which would, in turn, be related to the settings of the micro fluidic detection device and mainly the deposition time interval selected. The integrated areas for Cu and Pb in a mixed standard solution increased with longer deposition times in the range of 60 to 400 s. However, during the development of the integrated system, a deposition time of 200 s was chosen in view of the need for a short measurement time. It can be assumed that the limited time available for ion deposition might be responsible for observed discrepancies from the photometric method.

**Fig. 7:**
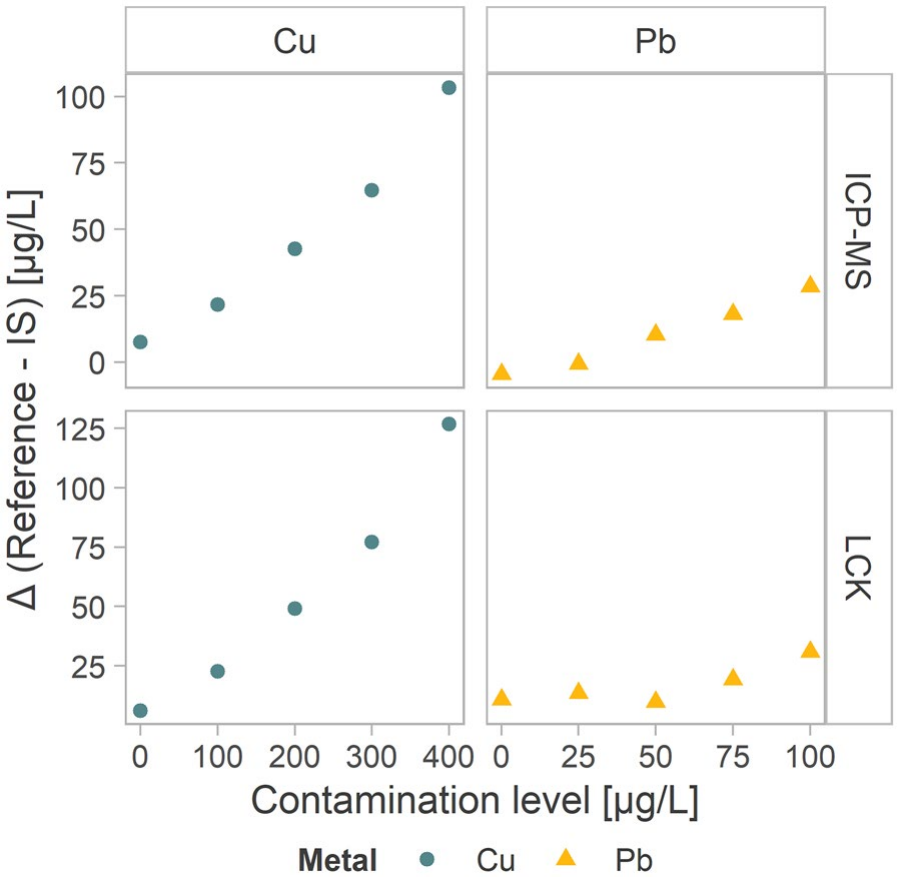
Difference between values measured by reference methods and the integrated system (IS) for each contamination level

The good comparability between ICP-MS and photometry (LCK) measurements is a strong indication of the limited formation of complexes between metal ions and humic substances. Therefore, the limited deposition capability of SWASV for metal-free ions may explain the bias between the integrated system and both reference methods. An additional confirmation was provided by the higher bias observed for higher (within the ranges used) ion concentrations for both metals and reference methods, as presented in Fig. 7.

This leads to the conclusion that the selected analytical methods cannot provide the agreed reference value needed for evaluation of bias in the electrochemical SWASV method used in this application. The authors decided to use the theoretical concentration value from the self-prepared solutions (control samples) for the evaluation of this characteristic.

### Validation through field experiments

**Fig. 8:**
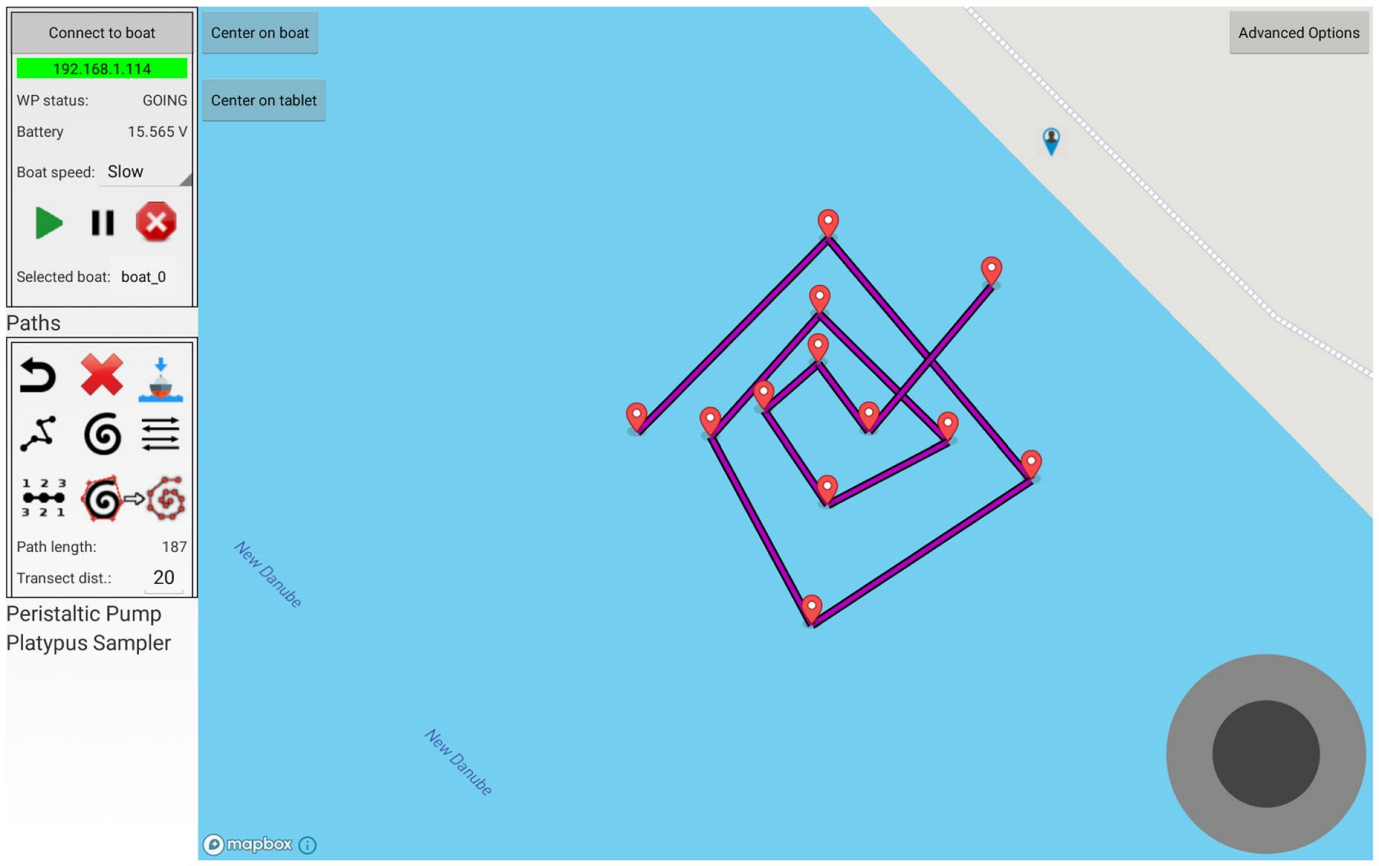
Graphical user interface and example of the pathway designed on the loaded live map of the selected site during one of the field campaigns

Three outdoor experiments were carried out to observe the behaviour and the performance of the integrated system under field conditions. The water quality of the selected catchment did not allow to observe any change in the concentrations of lead and copper during the measurement campaign. The total concentrations of Pb and Cu in all collected samples analysed by the ICP-MS method were below the limit of quantification. Nevertheless, during the outdoor experiments, it was possible to test the simultaneous operation of the autonomous features of the integrated system: the continuous measurement of the water matrix through the microfluidic device, the automatic collection of the water matrix through the sampler, and the autonomous navigation of the ASV. Several customized navigation paths were created with the use of the GUI, and the integrated system successfully responded to autonomous navigation by following the path created (Fig. 8).

The range of connectivity (between the integrated system and the GUI) was evaluated to be less than a 100-m radius. However, the lack of connection between the GUI and the autonomous surface vehicle, once the 100-m limit of radius was reached, did not influence the autonomous behaviour of the integrated system: the autonomous surface vehicle continued navigating, and the microfluidic system device kept performing the planned measurements. When the system was out of range from the GUI, only the functionality of the latter was affected: the operator cannot change parameters for the autonomous behaviours (i.e. change waypoints or path) and can control the boat only through a remote control (RC) unit. Future developments of the autonomous surface vehicle will include the use of 3G/4G technology to connect the GUI to the autonomous surface vehicle to avoid issues with the connectivity range. Furthermore, the performance of the HM detection device was tested throughout autonomous navigation. It was run in “continuous measurement” mode and showed that all measurements were successfully completed, and the results were saved in the BlueGate cloud and kept available for further computation. The sampling executed by the sampling device, which was implemented on the integrated system, was also successful. The vessels were filled independently and simultaneously after specific commands were used with the GUI.

The study from Wang et al. (2017) present a similar autonomous surface vehicle (kayak) equipped with an on-site Zn detection, using liquid crystal polymer-based electrodes coupled with SWASV. However, this autonomous monitoring device needed on shore commands, for the ASV’s navigation and for the performance of any electrochemical measurement, commands which are both automized on the integrated system of the present study (Wang et al., 2017).

### Validation results

The validation of the integrated system was partly based on the guidelines available in the ISO 15839 standard (International Standard, 2003, 2019). The performance characteristics of the system were computed by employing data collected during the laboratory experiments. As established in “Calibration function”, all selected data were calculated with cal2. The limit of detection (LOD), limit of quantification (LOQ) and lowest detectable change (LDC) are considered as the most important characteristics for this application. The recovery was used to represent the accuracy of the measurement. Finally, the measurement precision was described through day-to-day repeatability (reproducibility). The obtained results are listed in Table 2.

**Table 2:** Average values (AV) of the performance characteristics of the integrated system and number of experiments (*n*)

**Performance characteristic**		**Pb**		**Cu**	
	**Unit**	**AV**	***n***	**AV**	***n***
**Limit of detection**	**µg/L**	4	157	7	157
**Limit of quantification**	**µg/L**	14	157	22	157
**Lowest detectable change**	**µg/L**	4–5	77	6–7	81
**Recovery**	**%**	75	280	65	280
**Reproducibility**	**%**	11–18	109	6–10	70

Berho et al. (2009) and Bernalte et al. (2020) employed a similar heavy metal detection device as a monitoring tool for surface waters. The main differences were that the working electrode used in the study from Berho et al. (2009) was modified with the deposition of a mercury salt, and the electrodes used in the Bernalte et al. (2020) study were composed of gold (working and counter electrodes) and of silver (pseudo-reference electrode). In both studies of Behro et al. (2009) and Bernalte et al. (2020), the instrument comprehended only the SPE sensor coupled with a SWASV using a portable device; however, they were not implemented on an autonomous surface vehicle as in the present study. The overall precision of the integrated system showed value ranges similar to the reproducibility calculated in the study of Berho et al. (2009) (14–17% for Pb, 5–16% for Cu), but the limits of quantification obtained herein were 5–7 times higher than the values reported in the earlier study (2 µg/L and 5 µg/L for Pb and Cu, respectively). Similarly, a high difference between the limit of quantification of the present study and of the study of Bernalte et al. (2020) can be observed (7.3 and 5.1 µg/L for Pb and Cu, respectively). This discrepancy can be explained by differences in the working electrodes, by some differences among the SWASV set-ups and by the fact that some compromises had to be made during the integration of SWASV into the integrated system, as discussed in “Validation through laboratory experiments”. On the contrary, a comparison with the study from Tasić et al. (2020) shows a similar or slightly better detection performance of Pb with the integrated system (LOD of 6 µg/L and LOQ of 20 µg/L), and a less reproducible performance (day-to-day repeatability of 5%), than the laser-pyrolized paper electrodes coupled with SWASV. This can be due to the different electrode type and different voltammetry settings. Table S1 shows the comparison between the performance characteristics of the different devices.

The authors are aware that the LOQ values of the integrated system are not low enough to address the limits of environmental quality standards (EQS) established by the Water Framework Directive (EU, 2000, 2013). While no specification can be found there for Cu, the annual average EQS for lead and its compounds in inland surface waters is specified to be 0.02 µg/L, and the maximal allowable concentration is defined by 14 µg/L. Consequently, it can be concluded that the presented system is not suitable for monitoring and assessment of the chemical status of surface waters through surveillance monitoring programmes, as foreseen in the WFD. On the other hand, the values obtained for the lowest detectable change (4–5 and 6–7 µg/L for Pb and Cu, respectively), indicate that the developed integrated system is a promising tool for investigative monitoring according to the WFD i.e. to ascertain the impact of urban and/or accidental pollution sources in inland surface waters. To confirm this hypothesis, different scenarios were calculated by combining Pb and Cu emissions measured at certain points of the urban water cycle, such as effluent from conventional wastewater treatment plants (WWTPs), from combined sewer overflow (CSO) and stormwater runoff (Amann et al., 2019; Braun et al. (2020); Clara et al., 2009; Clara et al., 2020; Clara et al., 2014; Clara et al., 2017; Hohenblum et al., 2000; Lambert et al., 2014; Revitt et al., 2019; Slobodnik et al., 2018; Toshovski et al., 2020; Zoboli et al., 2019), and their possible dilutions into surface water receivers of different sizes (BMLRT, 2002; Department of Environment Food and Rural Affairs 2021; Wicke et al., 2015). For each studied heavy metal (Cu and Pb), 15 scenarios were considered: three emission pathways (CSO, stormwater runoff, WWTP) and five different urban situations for each of them, such as large urban area and large receiver (LL), large urban area and small receiver (LS), medium urban area and medium receiver (MM), medium urban area and small receiver (MS), small urban area and small receiver (SS)﻿.

**Fig. 9:**
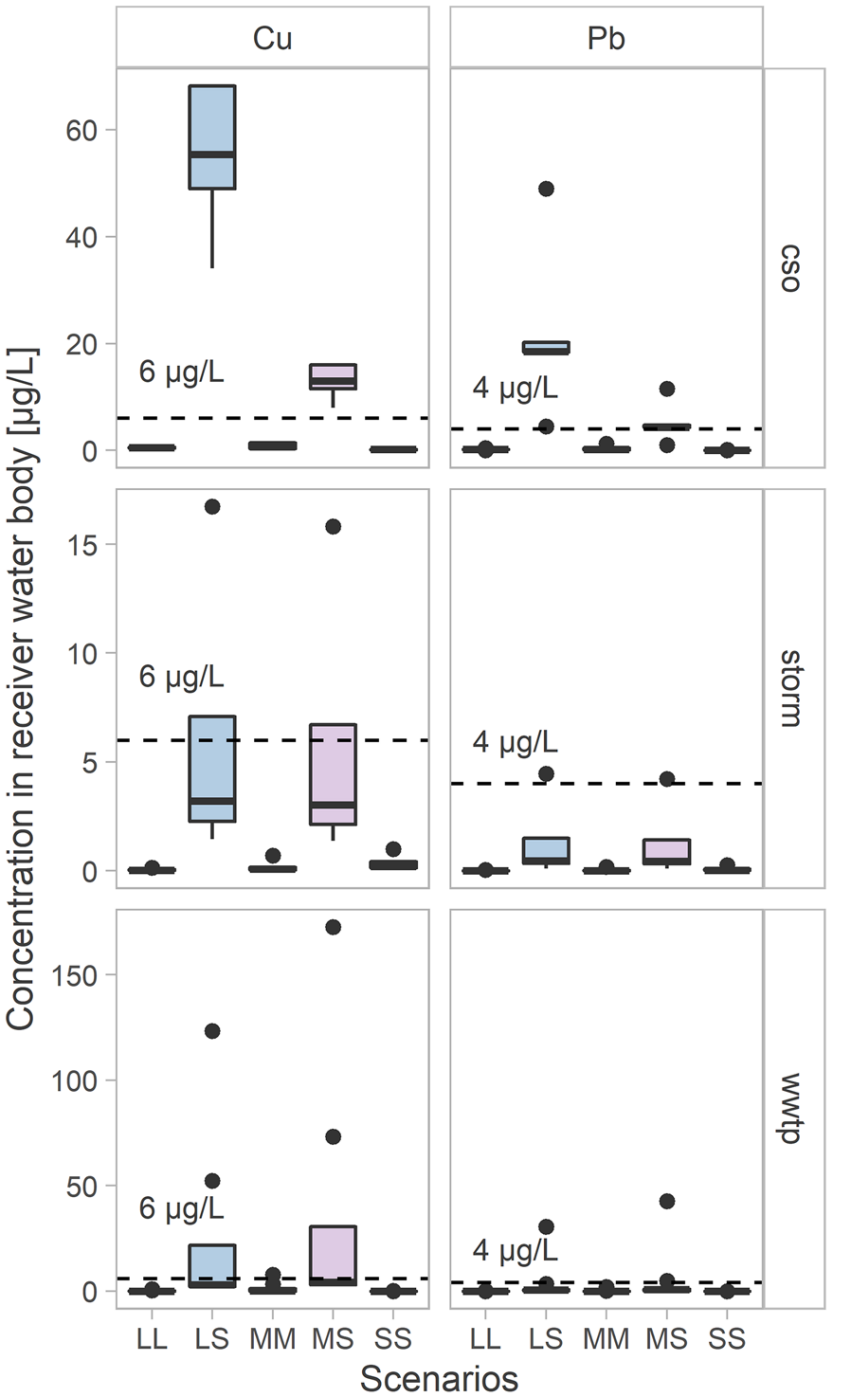
Potential concentrations of Cu and Pb in receiver water bodies for fifteen different scenarios: five urban area types (LL, LS, MM, MS, SS) for three output pathways (CSO, storm, WWTP). The dashed lines indicate the LDC values of the integrated system (6 µg/L and 4 µg/L for Cu and Pb, respectively)

As seen in Fig. 9, the lowest detectable change of the integrated system (dashed line) is suitable for detection of Cu and Pb pollution coming from CSOs in large and medium urban areas combined with a small receiver, which can cause concentrations up to 48 µg/L and 68 µg/L for Pb and Cu, respectively. The same conclusion can be reached for the detection of Cu pollution from CSOs and WWTPs because Cu comes from large and medium urban areas and is discharged in a small receiving water body, and the majority of the calculated concentration values are significantly higher than the obtained LDC (concentrations up to 16 µg/L and 173 µg/L for CSOs and WWTPs, respectively). Detection of Cu pollution from stormwater runoff can be considered possible but limited to extremely high pollution emissions. Furthermore, it can be assumed that the validated system could be appropriate to detect misconnections and cross connections in urban areas through on-site and real-time monitoring of the receiving waters. These pollution sources are known to induce high immediate emissions into receiving water, but no calculation was possible for this case because of the lack of data in the literature regarding Pb and Cu concentrations emitted. From the different combinations of concentrations, surface waters and pathways, it was possible to observe that the integrated system is fit-for-purpose in detecting pollution plumes in surface water bodies when a large or medium urban area discharges into a small receiver.

## Conclusions

An autonomous robotic system composed of a commercial autonomous surface vehicle equipped with additional features was developed to perform preprogrammed navigation in surface water bodies and simultaneous automatic sampling and chemical analysis. The architecture of the electronic system allows remote control of navigation, initialization of on-site measurements and collection of additional samples. The system is equipped with a microfluidic device, which allows in-line sampling, filtering, sample pretreatment and detection of heavy metals using square wave anodic stripping voltammetry and screen-printed electrodes. The miniaturized dimensions of components allow the implementation of an automated heavy metal detection device on a portable robotic system, enabling on-site investigative monitoring of source-point pollution in surface water.

As the calibration function strongly influences the final measurement accuracy, its role was first evaluated. In the case of using SPE as a sensing element, this influence is of crucial importance; the calibration function is related to the specific electrode in use, which needs to be changed for each measuring campaign. Three regression line trends were considered for the calibration function, and the forced-through-zero linear calibration function was selected for further calculations.

The lifetime of the sensor (maximal possible number of measurements) was evaluated, as it was considered a limiting factor for field monitoring campaigns. The average value of 39 measurements per sensor, which include a quality assurance test, the generation of a calibration function and effective on-site monitoring of the water body, was considered sufficient for the intended monitoring goals.

An extensive validation of the integrated system, partly based on ISO 15839 (International Standard, 2003, 2019), was performed to test its ability for on-site detection of heavy metal pollution plumes in river catchments. The main part of this validation process consisted of the simulation of pollution plumes under laboratory conditions. Field experiments were also performed at the Danube River to observe the behaviour and the performance of the integrated system under field conditions. The features, autonomous navigation, continuous measurement of the water matrix with the HM microfluidic detection device and autonomous sampling, worked properly in concert, and all responded to the commands given through the GUI. The measurement bias values, evaluated through recovery, were 75% and 65% for lead and copper, respectively (mean values from 280 measurements). Although these values are not very satisfactory, especially compared with those from reference laboratory analyses, they are considered acceptable for the purpose of this study, which is the detection of point pollution (i.e. severe concentration change occurring in a short time) rather than accurate measurement of HM concentrations in surface water. The measurement precision levels, evaluated as reproducibility and given as relative standard deviation (RSD in %), were 11–18% and 6–10% for Pb and Cu, respectively, which is considered satisfactory for on-site measuring systems. The estimated limit of detection (LOD) was rather poor (4 µg/L and 7 µg/L for Pb and Cu, respectively) for monitoring and assessing the chemical status of surface waters through surveillance monitoring programmes, according to the European Water Framework Directive. As the lowest detectable change (LDC) is considered the most important performance characteristic for the detection of pollution plumes in a water body, intensive experiments were carried out to estimate this performance characteristic. The estimated values for the lowest detectable change were 4–5 µg/L and 6–7 µg/L for Pb and Cu, respectively, which are considered satisfactory for detecting severe concentration gradients in a specific water body. To support this hypothesis, different scenarios for Pb and Cu emissions in urban areas were calculated for water body receivers of different sizes. The considered scenarios included three emission pathways (CSO, stormwater runoff, WWTP) and five urban area characteristics for each of them. It can be concluded that the developed and validated autonomous robotic system represents an innovative tool for investigative monitoring of aquatic environment and is considered fit-for-purpose for the detection of Pb and Cu emissions from large and medium urban areas that discharge into a small water body receiver.

Further studies are ongoing to improve the configuration and the performance characteristics of the developed integrated system, aiming to decrease detection limits, to improve the accuracy of multiple heavy metal detection and to widen the range of metals detected. The development of such a portable autonomous monitoring equipment is rather challenging, but a promising alternative to standard laboratory methods.

## Supplementary Information

Below is the link to the electronic supplementary material.

Supplementary file1 (DOCX 313 KB)

Supplementary file2 (JPG 3.16 MB)

## Data Availability

The datasets used and/or analysed during the current study are available from the corresponding author on reasonable request.

## References

[CR1] Amann, A., Clara, M., Gabriel, O., Hochedlinger, G., Humer, M., Humer, F., Kittlaus, S., Kulcsar, S., Scheffknecht, C., Trautvetter, H., Zessner, M., & Zoboli, O. (2019). STOBIMO Spurenstoffe, Stoffbilanzmodellierung für Spurenstoffe auf Einzugsgebietsebene. Wien.

[CR2] Barón-Jaimez J, Joya MR, Barba-Ortega J (2013). Anodic stripping voltammetry ASV for determination of heavy metals. Journal of Physics: Conference Series.

[CR3] Berho, C., Guigues, N., Ghestem, J. P., Crouzet, C., Strugeon, A., Roy, S., et al. (2009). On-site heavy metal monitoring using a portable screen-printed electrode sensor. In C. Gonzalez, R. Greenwood, & P. Quevauviller (Eds.), *Rapid chemical and biological techniques for water monitoring* (pp. 263–273, Water quality measurements series). Chichester, U.K.: Wiley.

[CR4] Bernalte E, Arévalo S, Pérez-Taborda J, Wenk J, Estrela P, Avila A (2020). Rapid and on-site simultaneous electrochemical detection of copper, lead and mercury in the Amazon river. Sensors and Actuators B: Chemical.

[CR5] BMLRT. (2002). eHYD Hydrographic yearbook interactive map. https://ehyd.gv.at/#. Accessed 24 March 2021.

[CR6] Boggs Jr, S., Livermore, D., & Seitz, M. G. (1985). Humic substances in natural waters and their complexation with trace metals and radionuclides: a review. [129 references]. (Report ANL-84-78, ON: DE85015539). United States. 10.2172/5569909

[CR7] Borrill AJ, Reily NE, Macpherson JV (2019). Addressing the practicalities of anodic stripping voltammetry for heavy metal detection: A tutorial review. The Analyst.

[CR8] Braun, R., Hartmann, C., Kreuzinger, N., Lenz, K., Schaar, H., & Scheffknecht, C. (2020). Untersuchung von Abwässern und Gewässern auf unterschiedliche toxikologische Endpunkte: Biologische Wirktests mittels in-vitro-Verfahren.

[CR9] Butterfield, M. (2009). Monitoring for heavy metals. *Air, Water, Environment International *(57).

[CR10] Clara, M., Denner, M., Gans, O., Scharf, S., Windhofer, G., & Zessner, M. (2009). Emissionen organischer und anorganischer Stoffe aus kommunalen Kläranlagen. (Report REP-0247,). Wien. http://www.umweltbundesamt.at/fileadmin/site/publikationen/REP0247.pdf

[CR11] Clara, M., Gruber, G., Hohenblum, P., Hofer, T., Kittlaus, S., Lenz, K., et al. (2020). TEMPEST: Erfassung von Emissionen ausgewählter Spurenstoffe aus Kanalsystemen, Handlungsoptionen zu deren Minderung und Opimierung einer alternativen Nachweismethode für Kunststoffpartikel in Wasserproben. Wien.

[CR12] Clara, M., Gruber, G., Humer, F., Hofer, T., Kretschmer, F., Ertl, T., et al. (2014). Spurenstoffemissionen aus Siedlungsgebieten und von Verkehrsflächen. SCHTURM. Wien. https://www.bmnt.gv.at/service/publikationen/wasser/Spurenstoffemissionen-aus-Siedlungsgebieten-und-von-Verkehrsflaechen.html

[CR13] Clara, M., Hanefeld, W., & Scheffknecht, C. (2017). Untersuchung ausgewählter prioritärer und sonstiger Stoffe in kommunalen Kläranlagen und Fließge-wässern in Vorarlberg Fließgewässern in Vorarlberg. https://vorarlberg.at/web/land-vorarlberg/contentdetailseite/-/asset_publisher/qA6AJ38txu0k/content/prioritaere-stoffe-in-kommunalen-klaeranlagen-und-fliessgewaessern?article_id=117972

[CR14] de la Escosura-Muñiz A, Ambrosi A, Merkoçi A (2008). Electrochemical analysis with nanoparticle-based biosystems. TrAC Trends in Analytical Chemistry.

[CR15] Department of Environment Food and Rural Affairs. (2021). Hydrology data explorer. Department of Environment Food and Rural Affairs. http://environment.data.gov.uk/hydrology/station/8496ce69-482c-406a-a2f0-ac418ef8f099

[CR16] EEA. (1996). European freshwater monitoring network design (10/1996).

[CR17] EEA. (2018). Environmental pressure of heavy metal releases from Europe’s industry (Industrial pollution in Europe No 3/2018). https://www.eea.europa.eu/publications/environmental-pressures-of-heavy-metal. Accessed 17 July 2020.

[CR18] EEA, Whalley, C., Mohaupt, V., Busch, W., van den Roovart, J., van Dujnhoven, N., et al. (2018). Chemicals in European waters: Knowledge developments (18/2018).

[CR19] EU. (2000). Directive 2000/60/EC of the European Parliament and of the Council of 23 October 2000 establishing a framework for community action in the field of water policy: WFD.

[CR20] EU. (2013). Directive 2013/39/EU of the European Parliament and of the Council of 12 August 2013 amending Directives 2000/60/EC and 2008/105/EC as regards priority substances in the field of water policy Text with EEA relevance: WFD.

[CR21] Gautam, R., Sharma, S., Mahiya, S., & Chattopadhyaya, M. (2014). Heavy metals in water: Presence, removal and safety.

[CR22] Gromaire MC, GARNAUD, S., Saad, M., & Chebbo, G. (2001). Contribution of different sources to the pollution of wet weather flows in combined sewers. Water Research.

[CR23] Güell R, Aragay G, Fontàs C, Anticó E, Merkoçi A (2008). Sensitive and stable monitoring of lead and cadmium in seawater using screen-printed electrode and electrochemical stripping analysis. Analytica Chimica Acta.

[CR24] Gumpu MB, Sethuraman S, Krishnan UM, Rayappan JBB (2015). A review on detection of heavy metal ions in water – An electrochemical approach. Sensors and Actuators B: Chemical.

[CR25] HACH. (2019). Copper trace cuvette test. https://uk.hach.com/copper-trace-cuvette-test-0-01-1-0-mg-l-cu-20-tests/product-details?id=26370291498. Accessed 9 January 2020.

[CR26] HACH. (2020). Lead cuvette test. https://uk.hach.com/lead-cuvette-test-0-1-2-0-mg-l-pb-25-tests/product-downloads?id=26370291402&callback=qs. Accessed 9 January 2020.

[CR27] Hohenblum P, Sattelberger R, Scharf S (2000). ABWASSER- UND KLÄRSCHLAMMUNTERSUCHUNGEN IN DER PILOTKLÄRANLAGEENTSORGUNGSBETRIEBE SIMMERING (EbS) (M-121).

[CR28] International Standard. (2003, 2019). Water quality - On-line sensors/analysing equipment for water: Specifications and performance tests (13.060.01, 13.060.45). Switzerland, 13.060.01, 13.060.45(15839). https://www.iso.org/standard/28740.html

[CR29] International Standard. (2012). Water quality - estimation of measurement uncertainty based on validation and quality control data (Vol. 13.060.45), 13.060.45(11352:2012). https://www.iso.org/standard/50399.html

[CR30] Jang A, Zou Z, Lee K-K, Chong AH, Bishop PL (2011). State-of-the-art lab chip sensors for environmental water monitoring. Measurement Science and Technology.

[CR31] Khan Y, Thielens A, Muin S, Ting J, Baumbauer C, Arias AC (2020). A new frontier of printed electronics: Flexible hybrid electronics. Advanced Materials.

[CR32] Koller, M., & Saleh, H. M. (Eds.). (2018). Introductory chapter: Introducing heavy metals. United Kingdom, London: IntechOpen.

[CR33] Lam MT, Chakrabarti CL, Cheng J, Pavski V (1997). Rotating disk electrode voltammetry/anodic stripping voltammetry for chemical speciation of lead and cadmium in freshwaters containing dissolved organic matter. Electroanalysis.

[CR34] Lambert, B., Fuchs, S., Toshovski, S., Sacher, F., & Thoma, A. (2014). Entwicklung eines Bilanzierungsinstruments für den Eintrag von Schadstoffen aus kommunalen Kläranlagen in Gewässer. Abschlussbericht gefördert unter dem Az: 29630 von der Deutschen. https://www.dbu.de/OPAC/ab/DBU-Abschlussbericht-AZ-29630.pdf. Accessed 6 May 2020.

[CR35] Law CS, Lim SY, Abell AD, Santos A (2018). Real-time binding monitoring between human blood proteins and heavy metal ions in nanoporous anodic alumina photonic crystals. Analytical Chemistry.

[CR36] Li M, Cao R, Nilghaz A, Guan L, Zhang X, Shen W (2015). “Periodic-table-style” paper device for monitoring heavy metals in water. Analytical Chemistry.

[CR37] Lu Y, Liang X, Niyungeko C, Zhou J, Xu J, Tian G (2018). A review of the identification and detection of heavy metal ions in the environment by voltammetry. Talanta.

[CR38] Masindi, V., & Muedi, K. L. (2018). Environmental contamination by heavy metals: 7. In H. E.-D. M. Saleh & R. F. Aglan (Eds.), Heavy metals (pp. 115–133). Rijeka: IntechOpen.

[CR39] Melo, M., Mota, F., Albuquerque, V., & Alexandria, A. (2019). Development of a Robotic Airboat for Online Water Quality Monitoring in Lakes (Robotics, Vol. 8).

[CR40] Merkoçi A, Vasjari M, Fàbregas E, Alegret S (2000). Determination of Pb and Cu by anodic stripping voltammetry using glassy carbon electrodes modified with mercury or mercury-nafion films. Microchimica Acta.

[CR41] Mohammed N, Baidya A, Murugesan V, Kumar AA, Ganayee MA, Mohanty JS (2016). Diffusion-controlled simultaneous sensing and scavenging of heavy metal ions in water using atomically precise cluster–cellulose nanocrystal composites. ACS Sustainable Chemistry & Engineering.

[CR42] Mostofa, K., Liu, C. Q., Mottaleb, M. A., Wan, G., Ogawa, H., Vione, D., et al. (2013a). Dissolved organic matter in natural waters. In (pp. 1–137).

[CR43] Mostofa, K. M. G., Liu, C. Q., Feng, X., Yoshioka, T., Vione, D., Pan, X., et al. (2013b). Complexation of dissolved organic matter with trace metal ions in natural waters. In K. M. G. Mostofa (Ed.), Photobiogeochemistry of organic matter: Principles and practices in water environments (Vol. 55, pp. 769–849, Environmental science and engineering. Environmental science). Heidelberg, New York: Springer.

[CR44] Mostofa KMG (2013). Photobiogeochemistry of organic matter: Principles and practices in water environments (Environmental science and engineering. Environmental science).

[CR45] Müller A, Österlund H, Nordqvist K, Marsalek J, Viklander M (2019). Building surface materials as sources of micropollutants in building runoff: A pilot study. The Science of the Total Environment.

[CR46] Odobašić, A., Šestan, I., & Begić, S. (Eds.). (2019). Biosensors for determination of heavy metals in waters.

[CR47] Palchetti I, Laschi S, Mascini M (2005). Miniaturised stripping-based carbon modified sensor for in field analysis of heavy metals. Analytica Chimica Acta.

[CR48] Pandey AK, Dhar Pandey S, Misra V (2000). Stability constants of metal–humic acid complexes and its role in environmental detoxification. Ecotoxicology and Environmental Safety.

[CR49] Pisarek I, Głowacki M (2015). Quality of groundwater and aquatic humic substances from main reservoire of Ground Water No. 333. Journal of Ecological Engineering.

[CR50] Rensing C, Maier RM (2003). Issues underlying use of biosensors to measure metal bioavailability. Ecotoxicology and Environmental Safety.

[CR51] Revitt, D. M., Ellis, J. B., & Lundy, L. (2019). Justification of an excel spreadsheet approach for predicting road runoff pollutant concentrations. Accessed 29 March 2021.

[CR52] Silva Junior, G. A., Lima Sa, T. S., Santos, H. D., Negreiros, P. Á., Souza Silva, M. J., Álvarez Jácobo, E. J., et al. (2016). Towards a real-time embedded system for water monitoring installed in a robotic sailboat (Sensors, Vol. 16). 10.3390/s16081226 PMC5017391 27509506

[CR53] Slobodnik J, Brack W, van Gils J, Dulio V, Liska I (2018). Guidance for identification of RBSPs and list of Danube RBSPs including quantification of their ecological impact and modelling-based exposure and risk predictions validated with case-study data. EU SOLUTIONS Project. External Deliverable.

[CR54] Steccanella, L., Bloisi, D., Blum, J., & Farinelli, A. (2019). Deep learning waterline detection for low-cost autonomous boats. In M. Strand, R. Dillmann, E. Menegatti, & S. Ghidoni (Eds.) (pp. 613–625). Cham: Springer International Publishing.

[CR55] Steinberg, C., Paul, A., Pflugmacher, S., Meinelt, T., Klöcking, R., & Wiegand, C. (2003). Pure humic substances have the potential to act as xenobiotic chemicals - A review. *Fresenius Environmental Bulletin,**12*, 391–401.

[CR56] Tasić N, de Sousa Oliveira L, Paixão TRLC, Moreira Gonçalves L (2020). Laser-pyrolysed paper electrodes for the square-wave anodic stripping voltammetric detection of lead. MEDICAL DEVICES & SENSORS.

[CR57] Toshovski, S., Kaiser, M., Fuchs, S., Sacher, F., Thoma, A., Kümmel, V., et al. (2020). Prioritäre Stoffe in kommunalen Kläranlagen: Ein deutschlandweit harmonisiertes Vorgehen (173). Dessau-Roßlau. https://www.umweltbundesamt.de/publikationen/prioritaere-stoffe-in-kommunalen-klaeranlagen

[CR58] Tuna G, Arkoc O, Gulez K (2013). Continuous monitoring of water quality using portable and low-cost approaches. International Journal of Distributed Sensor Networks.

[CR59] Verma, N., & Singh, M. (2005). Biosensors for heavy metals. *Biometals: An international journal on the role of metal ions in biology, biochemistry, and medicine, 18*, 121–129. 10.1007/s10534-004-5787-3. 10.1007/s10534-004-5787-3 15954738

[CR60] Waheed A, Mansha M, Ullah N (2018). Nanomaterials-based electrochemical detection of heavy metals in water: Current status, challenges and future direction. TrAC Trends in Analytical Chemistry.

[CR61] Wang N, Kanhere E, Kottapalli AGP, Miao J, Triantafyllou MS (2017). Flexible liquid crystal polymer-based electrochemical sensor for in-situ detection of zinc(II) in seawater. Microchimica Acta.

[CR62] Wicke, D., Matzinger, A., & Rouault P. (2015). Relevanz organischer Spurenstoffe im Regenwasserabfluss Berlins,

[CR63] Wiklund J, Karakoç A, Palko T, Yiğitler H, Ruttik K, Jäntti R (2021). A review on printed electronics: Fabrication methods, inks, substrates, applications and environmental impacts. Journal of Manufacturing and Materials Processing.

[CR64] Winters N, Granuke K, McCall M (2015). Roofing materials assessment: Investigation of five metals in runoff from roofing materials. Water Environment Research: A Research Publication of the Water Environment Federation.

[CR65] Winters, N. L., & Graunke, K. (2014). Roofing materials assessment: Investigation of toxic chemicals in roof runoff. *Washington State Department of Ecology Olympia, Washington, *98504–7710.

[CR66] Yang Q, Nagar B, Alvarez-Diduk R, Balsells M, Farinelli A, Bloisi D (2021). Development of a heavy metal sensing boat for automatic analysis in natural waters utilizing anodic stripping voltammetry. ACS ES&T Water.

[CR67] Zinoubi, K., Majdoub, H., Barhoumi, H., Boufi, S., Jaffrezic-Renault, N. (None). (2017). Determination of trace heavy metal ions by anodic stripping voltammetry using nanofibrillated cellulose modified electrode. *Journal of Electroanalytical Chemistry*, *799,* 70-77. 10.1016/j.jelechem.2017.05.039

[CR68] Zoboli O, Clara M, Gabriel O, Scheffknecht C, Humer M, Brielmann H (2019). Occurrence and levels of micropollutants across environmental and engineered compartments in Austria. Journal of Environmental Management.

