# Supplementary material for: An innovative autonomous robotic system for on-site detection of heavy metal pollution plumes in surface water
Source: Environ Monit Assess. 2022 Jan 24;194(2):122. doi: 10.1007/s10661-021-09738-z (PMC8786775; doi:10.1007/s10661-021-09738-z)
Supplement: Supplementary file 1 — Supplementary file1 (DOCX 313 KB) [file 10661_2021_9738_MOESM1_ESM.docx]

An innovative autonomous robotic system for on-site detection of heavy metal pollution plumes in surface water

Elisabetta De Vito-Francesco^a^, Alessandro Farinelli^b^, Qiuyue Yang^c,d^, Bhawna Nagar^c,e^, Ruslan Álvarez^c^, Arben Merkoçi^c,f^, Thorsten Knutz^g^, Alexander Haider^g^, Wolfgang Stach^a^, Falko Ziegenbalg^a^, Roza Allabashi^a^

^a^ University of Natural Resources and Life Sciences, Vienna (BOKU); Department of Water, Atmosphere, and Environment, Institute of Sanitary Engineering and Water Pollution Control, Muthgasse 18, 1190 Vienna, Austria

^b^ University of Verona, Department of Computer Science, Ca Vignal 2, Strada le Grazie 15, 7134, Verona, Italy

^c^ Catalan Institute of Nanoscience and Nanotechnology, UAB Campus, Bellaterra 08193, Barcelona, Spain

^d^ Materials Science, Department of Chemistry, Universitat Autònoma de Barcelona, Plaça Cívica, 08193 Bellaterra, Barcelona, Spain

^e^ Laboratory of Physical and Analytical Electrochemistry (LEPA), Ecole Polytechnique Fédérale de Lausanne (EPFL) Valais Wallis, Sion 1950, Switzerland

^f^.ICREA, Institució Catalana de Recerca i Estudis Avancats, Barcelona 08010, Spain

^g^Go Systemelektronik GmbH; Falunerweg 1, D-24109 Kiel, Germany

Corresponding author: De Vito-Francesco, Elisabetta; elisabetta.de-vito-francesco@boku.ac.at ; Muthgasse 18, 1190 Vienna, Austria; ORCID 0000-0003-1123-2336

# Supplementary Information


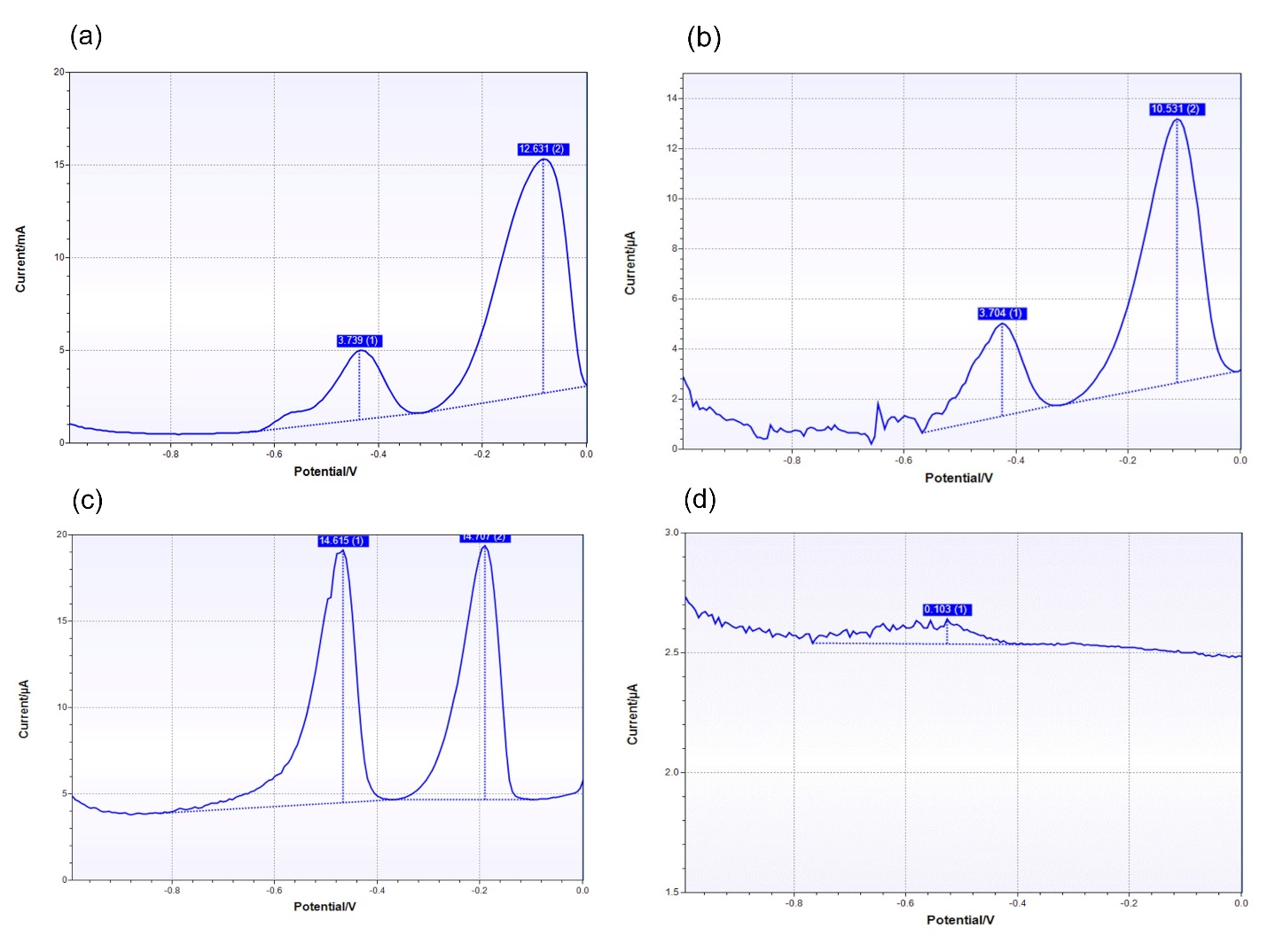


**Fig. S1** Representative voltammograms obtained after measurement process with the integrated system in different conditions: (a) laboratory measurement of pure water sample spiked with 50 and 200 µg/L for Pb and Cu respectively. The measured areas were 0.454 µAV (left peak) and 1.816 µAV (right peak) for Pb and Cu, respectively; (b) laboratory measurement in the water tank (500 L) filled with Danube water spiked with 25 µg/L and 100 µg/L for Pb and Cu respectively. The measured areas were 0.396 µAV (left peak) and 1.244 µAV (right peak) for Pb and Cu respectively; (c) laboratory measurement in the water tank (500 L) filled with ground water (bank filtration of Danube) spiked with 25 µg/L and 100 µg/L for Pb and Cu respectively. The measured areas were 1.46 µAV (left peak) and 1.27 µAV (right peak) for Pb and Cu respectively; (d) field measurement of the Danube River. The measured areas were 0.018 µAV for Pb and Cu was not detected (<LOQ).

**Table S1** Comparison of the performance characteristics among the studied integrated system and three other similar portable devices, using electrodes coupled with the SWASV electrochemical method.

|  | **Pb** |  |  | **Cu** |  |  |
| --- | --- | --- | --- | --- | --- | --- |
|  | **LOD [µg/L]** | **LOQ [µg/L]** | **Reproducibility %** | **LOD [µg/L]** | **LOQ [µg/L]** | **Reproducibility %** |
| **Integrated system** | 4 | 14 | 11 - 18 | 7 | 22 | 6 - 10 |
| **Berho et al. (2009)** | ­- | 2 | 14 - 17 | ­- | 5 | 5 - 16 |
| **Bernalte et al. (2020)** | 2.2 | 7.3 | ­- | 1.5 | 5.1 | ­- |
| **Tasic et al. (2020)** | 6 | 20 | 5 | ­- | -­ | -­ |

References

Berho C, Guigues N, Ghestem J-P, Crouzet C, Strugeon A, Roy S, Fouillac A-M (2009) On-Site Heavy Metal Monitoring Using a Portable Screen-Printed Electrode Sensor. In: Gonzalez C, Greenwood R, Quevauviller P (eds) Rapid chemical and biological techniques for water monitoring. Wiley, Chichester, U.K., pp 263–273

Bernalte E, Arévalo S, Pérez-Taborda J, Wenk J, Estrela P, Avila A, Di Lorenzo M (2020) Rapid and on-site simultaneous electrochemical detection of copper, lead and mercury in the Amazon river. Sensors and Actuators B: Chemical 307:127620. doi: 10.1016/j.snb.2019.127620

Tasić N, Sousa Oliveira L de, Paixão, Thiago R. L. C., Moreira Gonçalves L (2020) Laser-pyrolysed paper electrodes for the square-wave anodic stripping voltammetric detection of lead. MEDICAL DEVICES & SENSORS 3(6):e10115. doi: 10.1002/mds3.10115
