# Supplementary figures and images for: An innovative autonomous robotic system for on-site detection of heavy metal pollution plumes in surface water
Source: Environ Monit Assess. 2022 Jan 24;194(2):122. doi: 10.1007/s10661-021-09738-z (PMC8786775; doi:10.1007/s10661-021-09738-z)

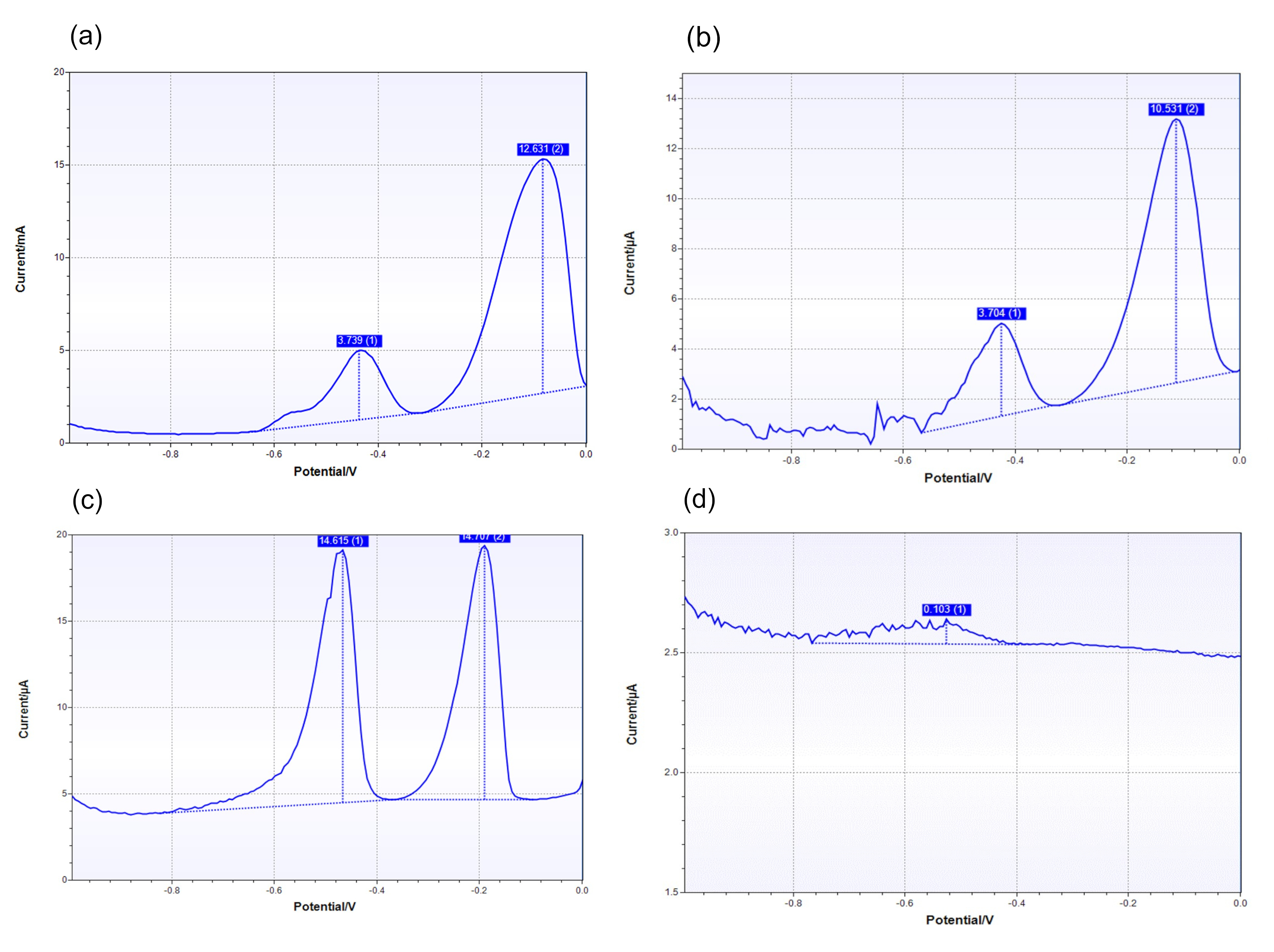

Supplement: Supplementary file 2 — Supplementary file2 (JPG 3.16 MB) [file 10661_2021_9738_MOESM2_ESM.jpg]
